# Tumor Mutation Burden Predicts Relapse in Papillary Thyroid Carcinoma With Changes in Genes and Immune Microenvironment

**DOI:** 10.3389/fendo.2021.674616

**Published:** 2021-06-23

**Authors:** Mengli Guo, Zhen Chen, Yayi Li, Sijin Li, Fei Shen, Xiaoxiong Gan, Jianhua Feng, Wensong Cai, Qingzhi Liu, Bo Xu

**Affiliations:** ^1^ Department of Thyroid Surgery, Guangzhou First People’s Hospital, Guangzhou Medical University, Guangzhou, China; ^2^ School of Medicine, South China University of Technology, Guangzhou, China; ^3^ Chronic Disease Laboratory, Institutes for Life Sciences and School of Medicine, South China University of Technology, Guangzhou, China

**Keywords:** papillary thyroid carcinoma (PTC), prognosis, The Cancer Genome Atlas (TCGA), tumor mutation burden (TMB), immune infiltrate

## Abstract

**Background:**

The risk factors of papillary thyroid carcinoma (PTC) recurrence are meaningful for patients and clinicians. Tumor mutation burden (TMB) has been a biomarker for the effectiveness of immune checkpoint inhibitor (ICI) and prognosis in cancer. However, the role of TMB and its latent significance with immune cell infiltration in PTC are still unclear. Herein, we aimed to explore the effect of TMB on PTC prognosis.

**Material and Methods:**

RNA-seq and DNA-seq datasets of PTC patients were downloaded from The Cancer Genome Atlas (TCGA) database. The Gene Ontology (GO) and gene set enrichment analysis (GSEA 4.0.1) were applied further to explore potential differences in PTC patients’ biological functions. The differentially expressed genes (DEGs) and immune microenvironment between the high and low TMB groups were determined.

**Results:**

TMB had the highest AUC score than other clinical indicators in ROC analysis on recurrence-free survival, and a higher TMB score was related to a worse prognosis. Further, GSEA showed a higher level of oxidative phosphorylation (OXPHOS) in the high TMB group, and four genes correlated with recurrence-free survival rate were identified. The abundance of CD8^+^ T cells and M1 macrophages in the high TMB group was significantly lower than that in the low TMB group.

**Conclusions:**

Our study found that TMB was a better predictor variable at evaluating the risk of PTC recurrence. Moreover, TMB-related genes conferred dramatically correlated prognosis, which was worth exploring in guiding postoperative follow-up and predicting recurrence for PTC patients.

## Introduction

Papillary thyroid carcinoma (PTC) is the most common pathological type of thyroid carcinoma, with a good prognosis (1). Over the last four decades the occurrence of thyroid cancer was growing for the increased incidence of PTC, and the increase in mortality of thyroid cancer was mainly due to the increase in mortality of advanced-stage PTC (2).

Although the recurrence rate of some low-risk and intermediate-risk PTC patients undergoing hemithyroidectomy is low, the current risk scoring system has no predictive effect on these patients, which needs to be followed up to detect and treat the recurrence early (3). The aggressive clinical pathological, age ≥55, male, tumor size >4 cm, tall cell variant, and positive lymph node metastasis are independent prognostic factors, and there may continue to be the risk of cancer death and early recurrence (4–7). Besides, many biomarkers for predicting the recurrence of PTC are reported, such as SMOC2 (8), Cyclin D1 and C-myc (9), serum fibrinogen levels (10), and BRAF^V600E^ mutation (11). However, most biomarkers have limitations or insufficient clinical applicability (12). It is imperative to find reliable prognostic markers for PTC.

Tumor mutation burden (TMB), which is a quantitative measurement of the total number of somatic non-synonymous mutations in each coding area of the tumor genome (13), was first reported to predict the activity of immune checkpoint inhibitor therapies in multiple cancers (14). The detection of TMB is based on massive next-generation sequencing (NGS) or whole-exome sequencing (15). In specific cancer types and ethnic groups, the number of driver mutations is significantly positively correlated with cancer incidence rates (16). One lung cancer research identified that specific mutations could result in tumorigenesis or recurrence, and patients with a high burden of tumor-specific gene mutations had poor recurrence-free survival (RFS) (17). Another study showed that further validation and research on the elevated TMB and the potential impact of tumor-initiating mutations in clinically unfavorable prostate cancer could improve the outcomes of patients (18). Higher TMB means more tumor neoantigens are exposed, so TMB has also become a predictor of response rate to immune checkpoint inhibitor therapies (19).

In this study, we investigated the features of TMB and explored the relationship of TMB with PTC recurrence. DEGs and immune cells were analyzed to explore the underlying mechanism of PTC recurrence.

## Methods

### Data Source

The RNA-seq and single nucleotide polymorphism (SNP) data were all obtained from the TCGA pan-cancer cohort of the GDC data portal (HTTPS://portal.gdc.cancer.gov/). The RNA-seq comprised 512 tumor samples and 58 normal samples. Out of 512 tumor samples, 487 had complete SNP data, including simple nucleotide variation data (VarScan) and masked somatic mutation data. Basic clinical information included age, gender, AJCC-TNM stages, pathological subtype, survival outcomes, and follow-up time. The “maftools” software package, which could convert mutation data in Variant Call Format (VCF) format into visualization results (20), was used to describe the basic mutation characteristics of PTC samples. The mutation information of genes in each sample was displayed in the waterfall chart.

### TMB Calculation

TMB generally refers to the number of somatic non-synonymous mutations per base pair (Mb) in a specific gene group region based on whole-exome modeling calculations. We selected SNP data from genome sequencing to calculate TMB. In our study, the mutation frequency and the number of variants in each sample were calculated through the Perl script based on the JAVA8 platform, as described in the previous literature (21). Then the PTC patients were divided into two groups according to the cut-off point (the median of the TMB value).

### Estimation of TMB and Prognostic Analysis

Then Kaplan–Meier analysis was utilized to compare the difference in RFS between the high and low TMB groups, evaluated by the log-rank test. Chi-square test was used to assess the differences of clinical characteristics between high and low TMB groups. Subsequently, the correlations of TMB levels with clinical characteristics were appraised. The Wilcoxon rank-sum test was used to compare the differences between the two groups of clinical variables. P-value <0.05 was considered statistically significant.

### DEGs and Functional Enrichment Analysis

DEGs were selected based on the high and low TMB groups of PTC patients through the “limma” software package (22), with log2 |fold change| >1 and False Discovery Rate (FDR) <0.05. The “heatmap” package was utilized to draw DEGs’ heatmap plot, and the “vioplot” package drew the volcano plot. Then we employed the Gene Ontology (GO) analyses through the “clusterProfiler” package (23) to analyze the possible biological processes of the overlapping DEGs. We also applied gene set enrichment analysis (GSEA 4.0.1) to further explore the potential differences in PTC patients’ biological functions.

### Estimation of Tumor-Infiltrating Immune Cells

We employed the downloaded RNA-seq data through the deconvolution algorithm in the CIBERSORT website (http://cibersort.stanford.edu/). CIBERSORT is a general calculation method that could combine support vector regression and prior knowledge of purified leukocyte subsets’ composition profile to accurately estimate the composition of 22 immune cells in tumor tissues, and the method could replace flow cytometry or immunohistochemistry which is difficult to standardize (24–26). We subsequently identified the differences of 22 immune cells between the two groups of PTC patients. “Vioplot” package was employed to show the differential immune infiltrates between the two groups. The data analysis used the Wilcoxon rank-sum test. The threshold p-value <0.05 was the criterion for calculating the significance of samples.

### Survival Analysis

Eighteen differential genes based on the two groups were identified. The RFS of these differential genes was analyzed according to the survival package of R software to find the more valuable prognostic biomarker. Receiver operating characteristic (ROC) and the area under the curve (AUC) were presented by utilizing the “survival ROC” package to confirm the prognostic value of clinical features. All data were tested by using an independent t-test, and the P-value was set to <0.05.

## Results

### Gene Mutation Profile of PTC

**Figure 1:**
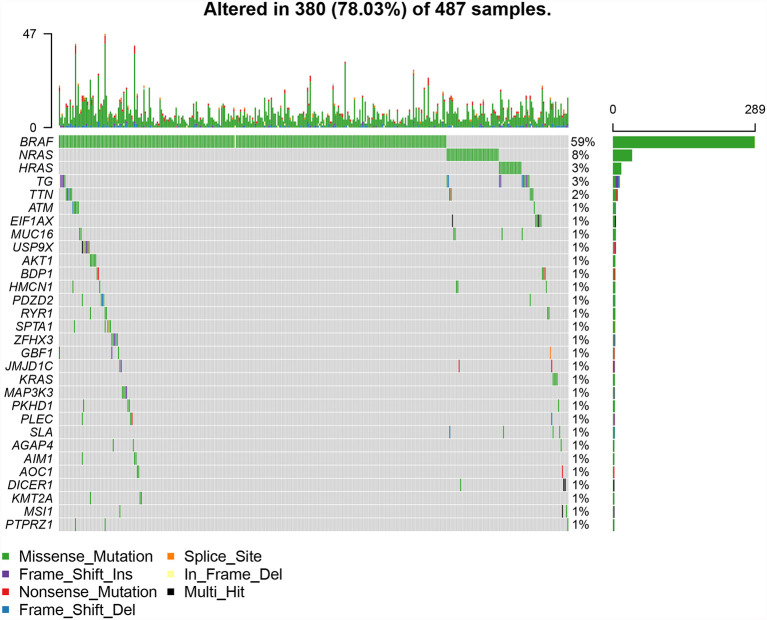
The landscape of most common mutated genes in PTC. The waterfall chart showed the top 30 frequently mutated genes of PTC in the TCGA cohort.

**Figure 2:**
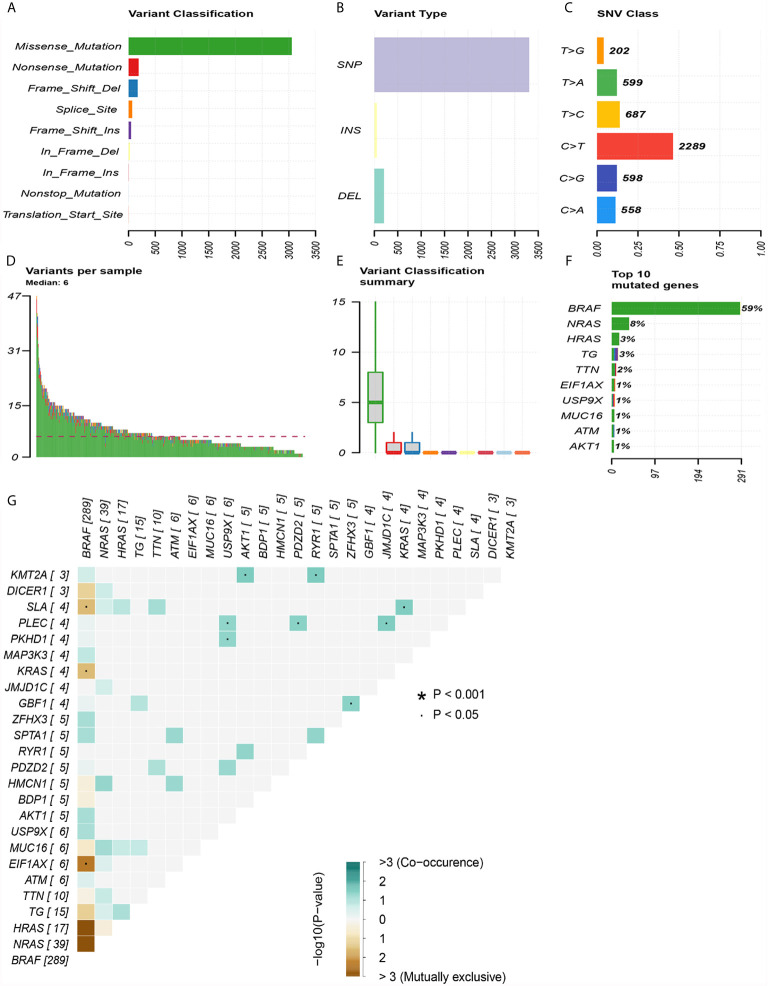
Summary of mutation information in the TCGA thyroid cancer cohort. **(A)** Types of gene mutations. **(B)** Types of genome variation. **(C)** Types of single nucleotide changes. **(D)** The median number of mutations per sample. **(E)** Summary of gene mutation categories. **(F)** Top 10 common mutant genes. **(G)** Coexistence and exclusivity between mutant genes.

The top 30 genes with a mutation frequency per sample were displayed, and different color annotations indicated the different types of mutations (**Figure 1**). The most common type of mutation in the summarized figure was a missense mutation (**Figure 2A**). SNPs occurred more often than insertions or deletions (**Figure 2B**). SNV was mainly based on C>T base substitution in PTC (**Figure 2C**). Also, we calculated the median of PTC variants per sample (**Figure 2D**) as six and summarized the variant classifications based on the median and range (**Figure 2E**). The top 10 most common mutated genes of PTC were exhibited (**Figure 2F**), in which BRAF mutations were observed in 59% of cases, NRAS mutations in 8%, HRAS mutations in 3%, TG mutations in 3%, and TTN mutations in 2%. Finally, we showed the coexistence and exclusive correlation between mutant genes, where green represented coexistence and brown described a mutually exclusive relationship (**Figure 2G**). Moreover, we could see the BRAF mutation had the exclusive association with NRAS mutation or HRAS mutation.

### The Differences in Different Clinical Characteristics Between the High and Low TMB Groups

We calculated the TMB of 487 PTC samples, which was the total number of mutations per million bases, ranging from 0.026 to 2.210 Mut/Mb, with a median of 0.211 Mut/Mb. According to the median value of TMB, we divided the PTC patients into the high and low TMB groups. Then we compared the differences in different clinical characteristics between the high and low TMB groups, including age, gender, AJCC-TNM stages, pathological subtype, BRAF status, and survival outcomes (**Table 1**). Because some samples lack clinical data, the number of samples exhibited in **Table 1** was not exactly complete. We could see that the high TMB group had a higher proportion of PTC patients with older than 55 years old, larger tumors, higher tumor stages, and higher recurrence rate.

**Table 1:** The differences of clinical characteristics between high and low TMB groups obtained from the TCGA cohort.

Clinical characteristic	Low TMB (%)	High TMB (%)	P value
**Age**			
<55	198 (40.8)	129 (26.6)	
>=55	45 (9.3)	113 (23.3)	**<0.001**
**Gender**			
Female	188 (38.8)	169 (34.8)	
Male	55 (11.3)	73 (15.1)	**0.06**
**AJCC-T**			
T1–2	167 (34.4)	130 (26.8)	
T3–4	76 (15.7)	111 (22.9)	**0.001**
TX	0 (0)	1 (0.2)	
**AJCC-N**			
N0	113 (23.3)	106 (21.9)	
N1	101 (20.9)	116 (23.9)	**0.291**
NX	29 (6.0)	20 (4.1)	
**AJCC-M**			
M0	138 (28,5)	135 (27.8)	
M1	3 (0.6)	6 (1.2)	**0.498**
MX	102 (21.0)	101 (20.8)	
**AJCC-Stage**			
Stages I–II	242 (49.9)	221 (45.6)	
Stages II–IV	1 (0.2)	21 (4.3)	**<0.001**
**Histopathology**			
Classical	175 (36.1)	165 (34.0)	
Follicular	52 (10.7)	48 (9.9)	
Tall Cell	13 (2.7)	23 (4.7)	**0.202**
Other	2 (0.4)	7 (1.4)	
**BRAF**			
Wild	102 (21.0)	94 (19.4)	
Mutation	140 (28.9)	149 (30.7)	**0.437**
**Outcome**			
Free-recurrence	221 (46.5)	209 (44)	
Recurrence	16 (3.4)	29 (6.1)	**0.043**

Bold values refers to whether there is a difference in the number of people with different clinical characteristics between the high TMB group and the low TMB group through the chi-square test, and a P value less than 0.05 has a statistical difference.

### Lower TMB Predicted Better RFS Rate in PTC

**Figure 3:**
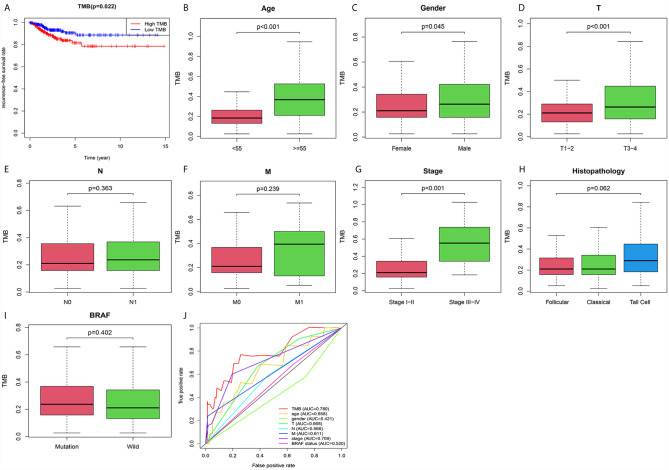
The prognostic value of TMB and its correlation with risky clinical features. **(A)** The association between TMB and recurrence-free survival rate. **(B)** Correlation between TMB and age. **(C)** Correlation between TMB and gender. **(D)** Correlation between TMB and AJCC-T staging. **(E)** Correlation between TMB and AJCC-N staging. **(F)** Correlation between TMB and AJCC-M staging. **(G)** Correlation between TMB and tumor staging. **(H)** Correlation between TMB and histopathology. **(I)** Correlation between TMB and BRAF status. **(J)** Confirming the predictive value of TMB and risky clinical features through the ROC curve.

Patients in the high TMB group tended to have a lower recurrence-free survival rate than that in the low TMB group (**Figure 3A**). In addition, TMB levels were higher in patients older than 55 (**Figure 3B**) or male sex (**Figure 3C**). PTC in the T3–4 stages had higher TMB levels than that in the T1–2 stages (**Figure 3D**). And TMB levels in stages III–IV were increased significantly compared with that in stages I–II (**Figure 3G**). However, levels of TMB grouped by AJCC-N (**Figure 3E**), M stage (**Figure 3F**), histopathological type (**Figure 3H**), and BRAF status (**Figure 3I**) had no significant differences. Most importantly, the ROC AUC score of TMB was 0.780, which was the most reliable indicator in the survival prediction of the model compared with age (AUC = 0.658), gender (AUC = 0.421), AJCC-T (AUC = 0.668), N (AUC = 0.566), M (AUC = 0.611), tumor stage (AUC = 0.708), or BRAF status (AUC = 0.520) (**Figure 3J**).

### Identification of DEGs Based on High and Low TMB Groups

**Figure 4:**
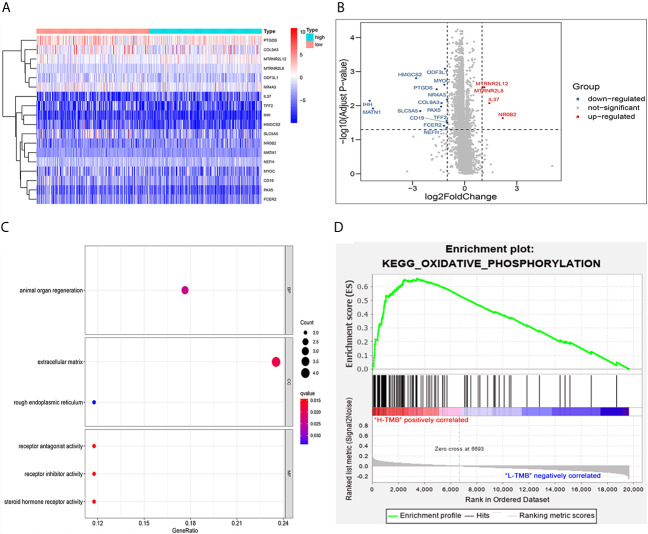
Analysis of differentially expressed genes and functional pathways in the low and high TMB groups. **(A)** Eighteen DEGs were exhibited in the heatmap. **(B)** Fourteen down-regulated and four up-regulated genes of high TMB group shown in volcano plot. **(C)** GO analysis of TMB-related DEGs in PTC. **(D)** Gene set enrichment analysis (GSEA) between high and low TMB groups (the high TMB group was positively correlated with OXPHOS, while the low TMB group was negatively correlated with OXPHOS).

Eighteen DEGs (**Table 2**) were identified from high and low TMB groups in the TCGA database, which were shown by heatmap (**Figure 4A**). Fourteen down-regulated and four up-regulated genes in high TMB group were shown in the volcano plot (**Figure 4B**). Further, we explored the function of DEGs by GO analysis and found that DEG enrichment was mainly animal organ regeneration, extracellular matrix, rough endoplasmic reticulum, receptor antagonist activity, receptor inhibitor activity, and steroid hormone receptor activity (**Figure 4C**). The GSEA result was related to oxidative phosphorylation (OXPHOS), which demonstrated that the TMB in the high TMB group was positively correlated with OXPHOS level, while that in the low TMB group was the opposite (**Figure 4D**).

**Table 2:** The DEGs between the high and low TMB groups.

Gene	Low group	High group	logFC	P Value	Fdr
MTRNR2L12	1.853415399	3.784501507	1.029917019	0.000119855	0.002855915
CD19	0.955320297	0.475328924	1.007058323	0.006612355	0.031412547
PTGDS	18.91732894	6.188140611	1.612130516	0.000154396	0.003304667
MATN1	2.635848637	0.068010736	5.276361212	0.001368037	0.012102619
MTRNR2L8	0.467813686	1.025258462	1.131981678	0.000122694	0.002880741
NEFH	0.655694924	0.318686632	1.040886222	0.011551044	0.0450017
NR4A3	3.455504029	1.706095324	1.018197905	0.000518091	0.00660994
TFF2	0.330944041	0.161350997	1.036384797	0.00559244	0.028075865
NR0B2	0.099958608	0.456218599	2.19032254	0.004161945	0.023153164
PAX5	0.316980166	0.123907105	1.355133659	0.001114339	0.010610705
COL9A3	34.47268561	13.70950704	1.330277001	0.000771706	0.008405303
MYOC	1.105746693	0.490957477	1.171350947	9.11E-05	0.002425095
SLC5A5	4.730948966	0.806373791	2.552608944	0.00189884	0.014405436
FCER2	0.532803227	0.233306308	1.191377504	0.009232025	0.038873341
IHH	0.598843568	0.013546866	5.466148113	0.000593825	0.007159124
IL37	0.134002953	0.358177823	1.418411225	0.000771791	0.008405303
HMGCS2	0.587826402	0.084715136	2.794698489	4.02E-05	0.00156174
ODF3L1	0.978796362	0.446031567	1.133862922	1.25E-05	0.000824246

### IL37, SLC5A5, NR4A3, and ODF3L1 Associated With PTC Relapse

**Figure 5:**
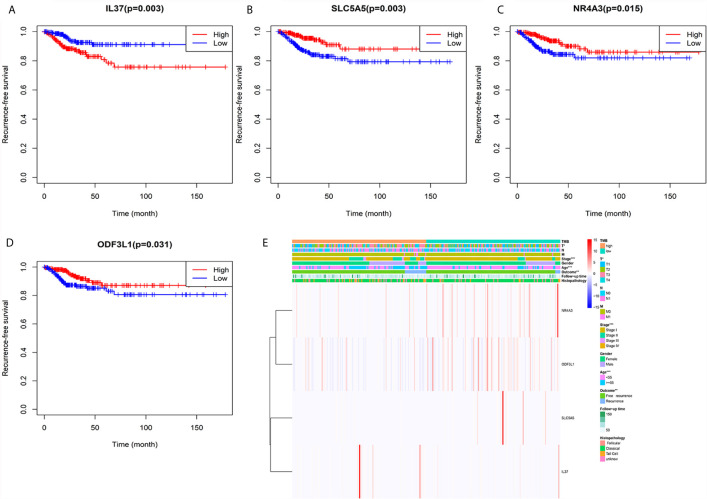
Heatmap of the relationship between TMB related genes and clinical features. **(A)** Kaplan–Meier survival analysis of IL37. **(B)** Kaplan–Meier survival analysis of SLC5A5. **(C)** Kaplan–Meier survival analysis of NR4A3. **(D)** Kaplan–Meier survival analysis of ODF3L1. **(E)** The expression levels of the four genes and distribution of clinical features between the high and low TMB groups.

**Figure 6:**
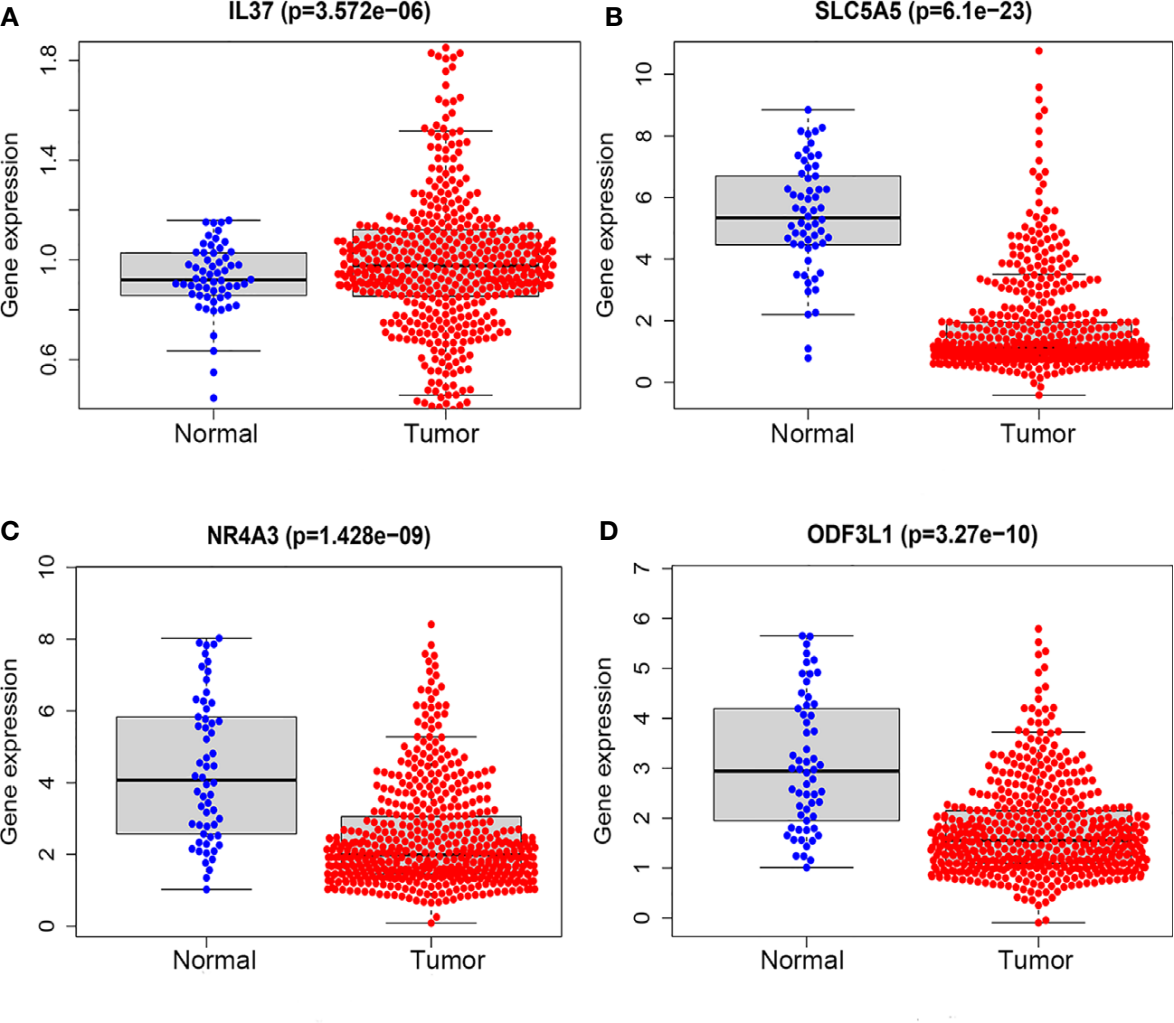
TMB related prognostic genes in tumor and normal tissues of PTC based on TCGA database. **(A)** IL37. **(B)** SLC5A5. **(C)** NR4A3. **(D)** ODF3L1.

We performed survival analysis on the 18 TMB related DEGs. Interleukin 37 (IL37), solute carrier family 5 member (SLC5A5), nuclear receptor subfamily 4 group A member 3 (NR4A3) and outer dense fiber of sperm tails 3 like 1 (ODF3L1) had been determined to be correlated with RFS of PTC patients. The results showed that higher expression of IL37 in tumor tissues predicted worse prognosis outcomes (**Figure 5A**). Conversely, the other three genes with a higher level in tumor tissues predicted a better prognosis (**Figures 5B–D**). The relationship between the four DEGs and each clinicopathological factor per sample in the high and low TMB groups was shown by the heatmap (**Figure 5E**). We could observe that the high expression of IL37 was concentrated in clinical features with poor prognosis, while the SLC5A5, NR4A3, and ODF3L1 were concentrated in clinical features with good prognosis. We further confirmed the expression levels of the four differential expression genes according to the RNA-sequencing dataset from TCGA. The expression level of IL37 in the tumor was significantly higher than that in the normal tissues (**Figure 6A**), while SLC5A5, NR4A3, and ODF3L1 were highly expressed in normal tissues than those in tumors (**Figures 6B–D**).

### Differences in Immune Cell Infiltration Between High and Low TMB Groups

**Figure 7:**
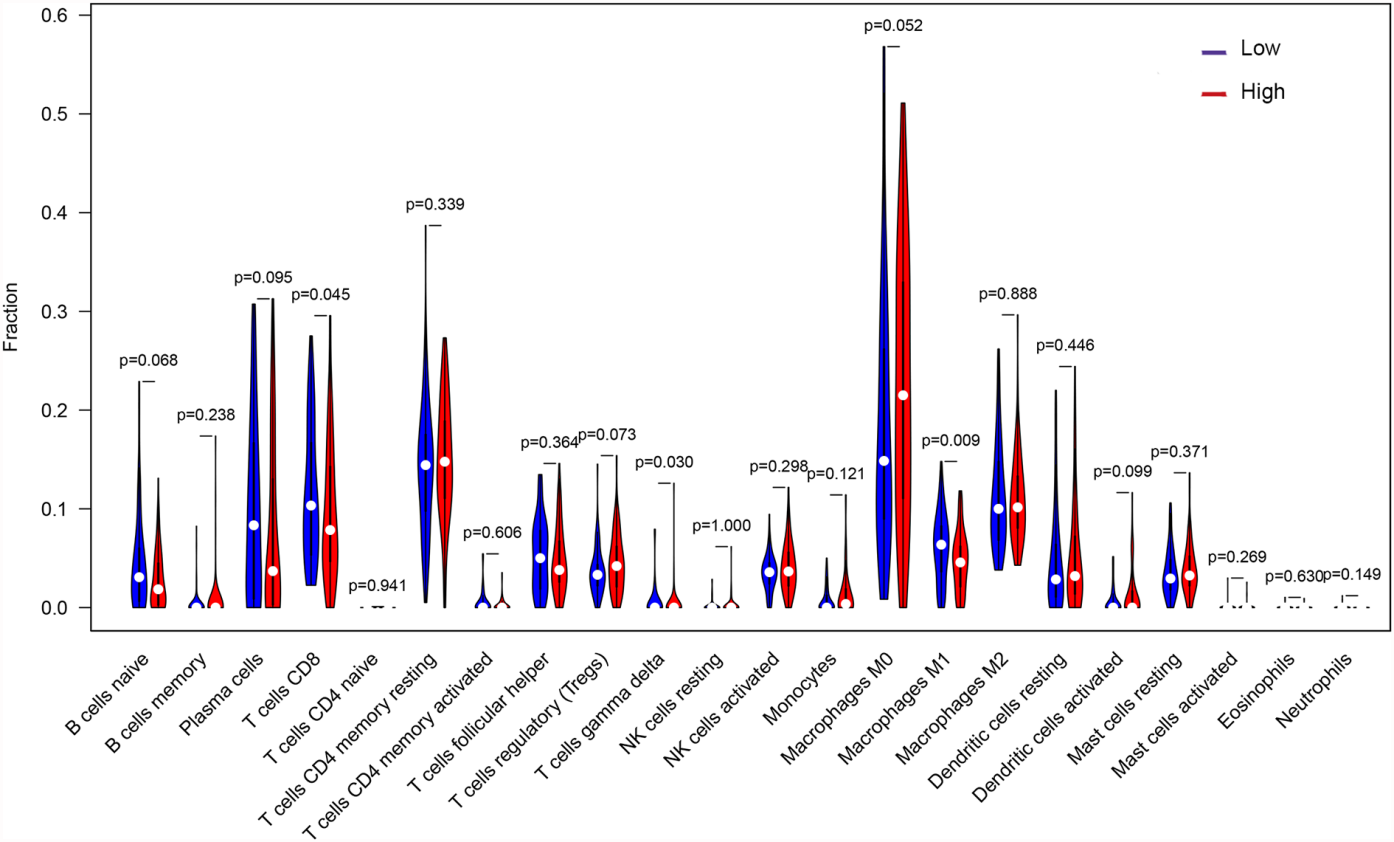
Abundance of 22 immune cell infiltration between the high and low TMB groups.

Through the deconvolution algorithm on the CIBERSORT website, the relative scale of 22 immune cells was measured. PTC patients in the high TMB group had lower infiltration of CD8^+^ T cells and M1 macrophages but higher gamma delta T cells than those in the low TMB group (**Figure 7**).

## Discussion

The previous study has shown that the Tumor Node Metastasis (TNM) staging guide cannot effectively predict the prognosis of patients with low-risk PTC during initial treatment (27). Herein, we defined the characteristics of TMB in PTC and compared TMB as the best recurrence predictor than other clinical features. Generally speaking, TMB is the number of somatic non-synonymous mutations per base pair (Mb) in a specific gene group region. Herein we selected SNP data which was similar to whole-exome sequencing to calculate TMB. However, the TMB values of patients with different cancer types are highly heterogeneous, and the high TMB thresholds screened out still show huge differences in different cancer types (28). Besides, since many of the original studies on TMB are based on whole-exome modeling calculations, it is usually visualized as the number of mutations in the coding region of the genome (exome) (29). In fact, in addition to point mutations that could produce neoantigens, frameshift mutations produced by insertions and deletions could also cause tumors to produce neoantigens, thereby changing the efficacy of immune checkpoint inhibitors (30). The TMB we calculated based on genome sequencing was caused by somatic non-synonymous mutations, and the changes in TMB which might be caused by other factors were not taken into account, such as insertions or deletions. Therefore, in future, TMB detection still needs to consider many other interference factors.

GO analysis revealed that DEG enrichment was mainly involved in metabolic pathway. Also, patients in high TMB group showed poorer RFS for an increase in the level of OXPHOS. CD8^+^ T cells and M1 macrophages were decreased, which might be responsible for the recurrence of PTC with high TMB. Together these findings indicated that TMB in the tumor could help in effectively taking initial measures to monitor recurrence probability and avoid secondary surgery in the clinic. TMB has been reported to play important roles in many cancers (31). Higher TMB has more favorable RFS and is also a good predictor of response to ICPIs within a high microsatellite instability population in colorectal cancer (32, 33). Conversely, our study showed that PTC patients in the high TMB group had worse RFS (**Figure 3A**). Higher TMB was also associated with age older than 55, male, tumor size, and tumor staging (**Figures 3B–D, G** **)**. The result was consistent with previous reports on clear cell renal cell carcinoma (21, 34). TMB had a rising trend in tall cell subtypes compared to classic and follicular types, but there might be no statistical difference due to insufficient sample size. BRAF^V600E^ mutation is significantly related to aggressive clinicopathological features and PTC relapse (35, 36). Furthermore, we also showed BRAF mutations as the most frequent and more prevalent in PTC than other mutations based on the TCGA data (**Figure 1**), which was consistent with previous reports (37). The interaction analysis of mutant genes showed that coexistence was more common than mutual exclusion (**Figure 2G**). However, compared with BRAF status only, TMB was a better predictor of relapse (**Figure 3H**). In general, ROC analysis showed the sensitivity and specificity of TMB were better than other clinical features. In our study, we found a significant risk factor for PTC recurrence and aggressiveness.

OXPHOS is up-regulated in many cancers, and its inhibitors with appropriate therapeutic index have new applications in targeted cancer therapy, including thyroid cancer (38–40). GSEA results showed higher levels of OXPHOS in the tumors of patients in the high TMB group (**Figure 4D**), which might be a potential relapse mechanism in PTC with a higher mutation burden. In the immune microenvironment of the tumor, CD8^+^ T cells can recognize neoantigens on the surface of tumor to play an anti-tumor effect (41). Besides, high CD8^+^ T cell density was significantly associated with a good disease-free survival rate for PTC patients and with a reduced lymph node metastasis incidence (42), and M1 macrophages in the tumor microenvironment inhibit tumor growth (43–46). The low abundance of CD8^+^ T cells and M1 macrophages was also found in PTC with high TMB (**Figure 7**), which might also clarify the underlying reasons of high TMB causing relapse.

Furthermore, four TMB related differential genes were highly associated with prognosis (**Figure 5**). IL37 was a risky prognostic gene and might play a vital role in the development of PTC. The result was consistent with oral leukoplakia and oral squamous cell carcinoma (47). However, IL37 plays a protective role in most cancers, such as lung cancer (48) and colorectal cancer (49). At present, the function of IL37 in the carcinogenesis of PTC has not been explored. SLC5A5, which codifies sodium iodide symporter, plays an essential role in thyroid metabolism, mediating the active transport of iodine from the bloodstream into the follicular cells (50). Due to the decreased expression of SLC5A5, some patients with differentiated thyroid cancer were not sensitive to radioiodine therapy (51). NR4A3 is an orphan receptor and regulates the cellular function and inflammation reaction (52). Compared with thyroid follicular adenoma (FTA), NR4A3 was significantly down-regulated in follicular thyroid carcinoma (FTC), which led to reduced apoptosis factors (53). However, the function of NR4A3 in PTC is still unclear. As for ODF3L1, there is no research report yet; however, it is an excellent prognostic molecular marker in PTC. Though we detected four genes associated with PTC relapse, these genes’ protein expression levels still need to be verified.

In conclusion, our results provided new information for the function of TMB in PTC. We showed that TMB was an independent prognostic factor to predict the recurrence of PTC and explained the possible mechanism. We also screened out genes related to relapse in PTC patients, which could become a new prognostic marker. At present, studies have shown that the targeted sequencing panel combined with bioinformatics algorithm could effectively reduce the detection cost of TMB and sample size requirements (54). Nevertheless, our study was retrospective based on public databases, and the sample size was not large enough. The detailed mechanism needs to be further elucidated in future studies. In general, our findings strongly suggest that TMB is clinically meaningful for monitoring the recurrence to shorten the follow-up time of PTC.

## Data Availability Statement

The datasets presented in this study can be found in online repositories. The names of the repository/repositories and accession number(s) can be found below: https://portal.gdc.cancer.gov/exploration.

## Ethics Statement

All data of the study were obtained from The Cancer Genome Atlas (TCGA) database and have obtained ethical approval.

## Author Contributions

MG, ZC, and BX conceived the study. QL, FS, and JF designed and revised the manuscript. MG, YL, SL, XG, FS, JF and WC analyzed the data. All authors contributed to the article and approved the submitted version.

## Funding

This research was supported by Guangzhou medicine and healthcare technology projects (20211A011010) and Guangzhou Science and Technology Plan Project (202102080170).

## Conflict of Interest

The authors declare that the research was conducted in the absence of any commercial or financial relationships that could be construed as a potential conflict of interest.
